# Focused attention meditation changes the boundary and configuration of functional networks in the brain

**DOI:** 10.1038/s41598-020-75396-9

**Published:** 2020-10-28

**Authors:** Shogo Kajimura, Naoki Masuda, Johnny King L. Lau, Kou Murayama

**Affiliations:** 1 Faculty of Information and Human Science, Kyoto Institute of Technology, 1, Matsugasakihashigami-cho, Sakyo-ku, Kyoto-shi, Kyoto, 606-8585 Japan; 2 Department of Mathematics, University at Buffalo, State University of New York, Buffalo, USA; 3 Department of Psychology, University of Reading, Reading, UK

**Keywords:** Neuroscience, Cognitive neuroscience

## Abstract

Research has shown that focused attention meditation not only improves our cognitive and motivational functioning (e.g., attention, mental health), it influences the way our brain networks [e.g., default mode network (DMN), fronto-parietal network (FPN), and sensory-motor network (SMN)] function and operate. However, surprisingly little attention has been paid to the possibility that meditation alters the architecture (composition) of these functional brain networks. Here, using a single-case experimental design with intensive longitudinal data, we examined the effect of mediation practice on intra-individual changes in the composition of whole-brain networks. The results showed that meditation (1) changed the community size (with a number of regions in the FPN being merged into the DMN after meditation) and (2) led to instability in the community allegiance of the regions in the FPN. These results suggest that, in addition to altering specific functional connectivity, meditation leads to reconfiguration of whole-brain network architecture. The reconfiguration of community architecture in the brain provides fruitful information about the neural mechanisms of meditation.

## Introduction

Meditation is a practice aimed to enhance one’s core psychological capacities, such as attentional and emotional self-regulation^1^. In several styles of practice, focused attention meditation involves sustaining attention to present-moment experiences without emotional reaction and judgment and has been found to produce significant beneficial outcomes, such as stress reduction^2^ and improvements in attention processing^3^.

Past research indicates that the meditation is primarily related to three brain networks: the fronto-parietal network (FPN), sensory-motor network (SMN), and default mode network (DMN)^1^. The FPN mainly consists of the rostro- and dorso-lateral prefrontal cortex (PFC), anterior insula, dorsal anterior cingulate cortex (ACC), and anterior inferior parietal lobule; all of these brain areas are critical for cognitive control functions, such as regulation of attention and emotion^4–7^. Especially in the early stages of long-term practice, this meditation increases activation of FPN regions^8–11^, which is consistent with the general observation that focusing on the present moment requires effortful attentional control.

Focused attention meditation also alters sensory experiences through the SMN^12,13^, consisting of motor cortices, primary somatosensory cortex, and insula. In a previous study, these brain areas showed reduced activation in a four-day meditation when beginners meditated in the presence of noxious stimulation causing pain^14^. This change in brain activity may be associated with enhanced body awareness, as the meditation requires individuals to focus on a body part or internal experiences, such as breathing^15^.

The DMN, mainly consisting of the anterior medial PFC, posterior cingulate cortex (PCC), and posterior inferior parietal lobule, is a network implicated in supporting spontaneous thoughts and self-referential processing^16–18^. Because sustained attention on an anchoring object (e.g., one’s breath) needs to detect distraction such as task-irrelevant thoughts, disengage attention from the distraction, and redirect attention on the object, the DMN is expected to be suppressed during meditation. In fact, the medial PFC and PCC showed less activity during meditation, and functional connectivity between the PCC, dorsal ACC, and dorso-lateral PFC was stronger in meditators compared to meditation-naive controls^19^. These results indicate that the meditation may increase cognitive control over the DMN functioning^19^.

Although previous work has provided various insights into how meditation influences the functional network of the brain, there are two critical limitations in the current literature. First, the brain networks were defined a priori in previous studies, precluding the possibility that meditation practice can alter the architecture of the primary brain networks themselves (i.e. FPN, SMN, and DMN). Because recent studies have shown that meditation can change functional connectivity across brain regions^10,19–21^, the whole-brain composition of the FPN, SMN, and DMN may be altered as a consequence of meditation.

Second, most of the previous research has employed a one-shot pre-post or nonmeditator-meditator comparison design^22,23^, and compared the conditions after aggregating the data across heterogeneous participants. This inter-individual aggregation approach is useful to examine the effects of meditation averaged across participants. However, given the large individual differences in the whole-brain functional connectivity patterns^24,25^, there is danger that the approach potentially masks important intra-individual changes in the composition of the brain networks (e.g., some participant-specific network architectures may be canceled out by inter-individual aggregation). Therefore, adopting a design that allows us to focus on the intra-individual change may provide novel insights into how meditation alters the architecture of the brain networks.

The current research aims to expand our understanding of meditation by addressing these two critical issues. For that purpose, we will examine the effects of meditation using a single-case experimental design with intensive longitudinal data. Single-case experimental designs have a long tradition in psychology (Fechner et al.^26^; Watson^27^), and in later years, they have been applied to intensive longitudinal data (for a systematic review, see Smith^28^). Single-case experimental designs are effective in reliably detecting intra-individual changes in outcome variables in response to intervention^28^. However, this design has rarely been implemented in neuroimaging studies (for an exception without experimental manipulation, see Poldrack et al.^29^). Based on this design, we scanned a single participant repeatedly over a long period of time (65 days), employing the meditation intermittently, and examined whether and how the whole-brain composition of the FPN, SMN, and DMN were altered over this period of meditation practice.

## Methods

The study was conducted in accordance with the principles of the Helsinki declaration.

### Participant

The participant (author S.K.) is a right-handed Asian male, aged 28 years and had no experience of meditation practice at the onset of the study. The participant was healthy with no history of neuropsychiatric disorders. The study was approved by the research ethics committees of the University of Reading, UK (UREC 16/28).

**Figure 1:**
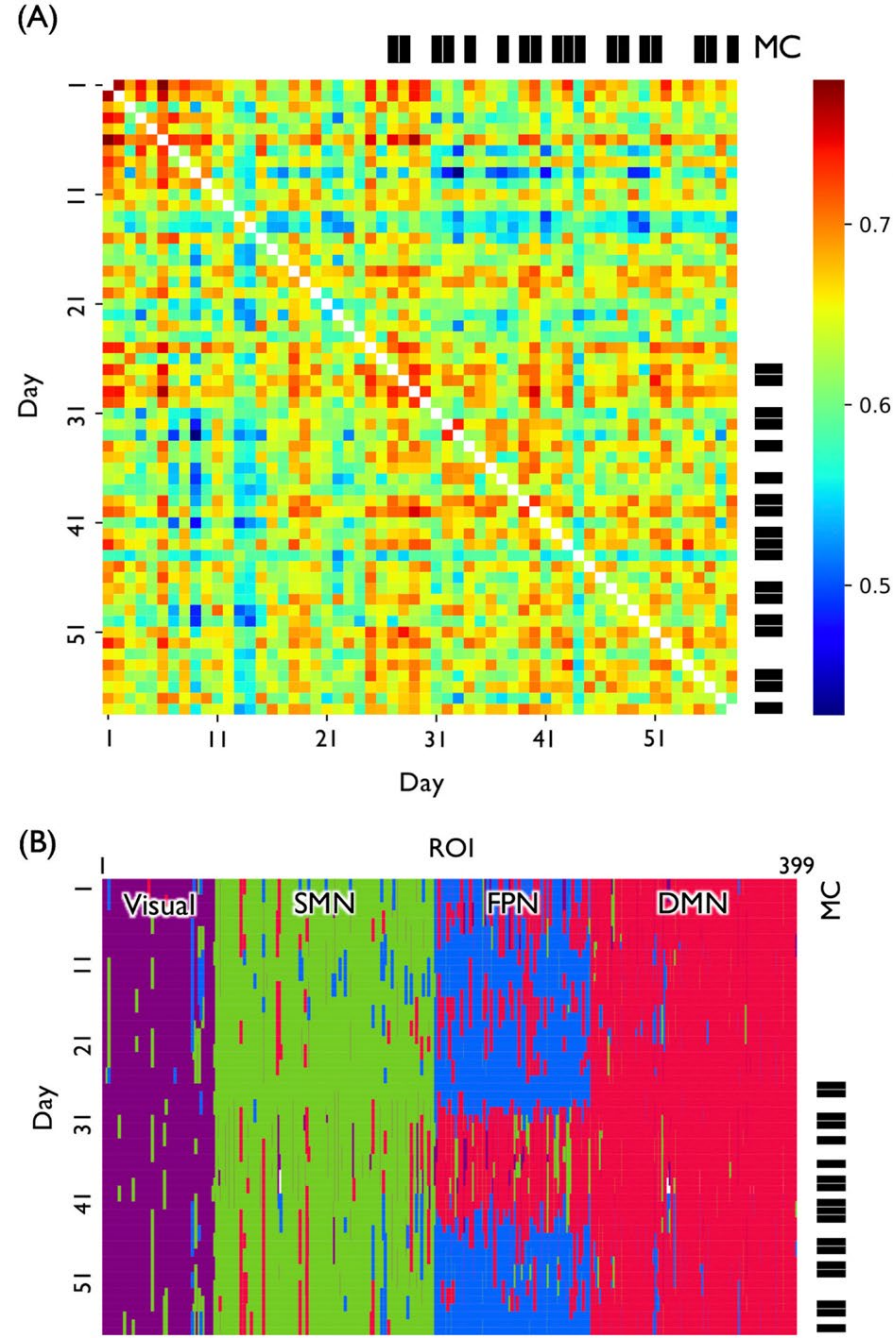
Properties of the ROIs that changes across days. (**A**) Similarity (correlation) between the functional networks for each pair of days. The correlation value ranged from 0.429 to 0.780, suggesting that the functional connectivity of a single person varies on a daily basis. The color code represents the value of the correlation coefficient. MC refers to the meditation condition. There were 18 out of 58 days in the MC. (**B**) Time-dependent community architecture. The rows and columns correspond to the days and ROIs, respectively. While the majority of the ROIs were classified to the same community, the ROIs in fronto-parietal network were often categorized as part of the default mode network. The four communities were labeled the visual network (colored in purple), sensory-motor network (SMN; green), fronto-parietal network (FPN; blue), and default mode network (DMN; red). A white stripe describes a day in which a ROI belonged to more than one community and could not be assigned a community label.

The participant underwent 65 scanning sessions, each on a different day between June 15th 2016 and November 11th 2016. The scanning time (between 9am and 5 pm) varied unsystematically across days. Data acquisition was not completed on 6 out of the 65 days due to technical issues and data from an additional day was excluded due to excessive head movement (> 3.0 mm between adjacent scan slices). As a result, only data from 58 days were used in the following analyses. On each day, the participant underwent a resting-state fMRI scan with eyes open for 10 min and completed two sets of questionnaires carried out for a separate study. On 18 out of the 58 days (Fig. 1), the participant underwent a 15-min session of the meditation practice within half an hour before the scanning session. Note that we started the intervention 64 days after the first scanning session and the assignment of the intervention condition was randomized since then. During the meditation, the participant was instructed to focus on his breathing, specifically on sensations of the breath through the nostrils, and to redirect attention from spontaneously occurring thoughts to breathing when he realized his mind was wandered^10^. Before the data collection, the participant studied the meditation several times from an auditory instruction developed by a professional trainer (Fujino et al.^30^, in revision) so that he did not need the instruction for each practice. In the following text, the “meditation condition” refers to the days on which scanning followed the meditation practice. The “no-meditation condition” refers to the days on which there was no meditation practice prior to scanning. The mean of recording days (i.e. days elapsed from the first scanning session) of the meditation condition and no-meditation condition were 23.7 and 42.3, respectively.

### Data acquisition

MR images were acquired using a Siemens 3.0-T Trio scanner equipped with a 32-channel head coil at the Centre for Integrative Neuroscience and Neurodynamics of the University of Reading. The resting-state fMRI data were obtained using a single-shot, gradient-echo echo-planar imaging (EPI) sequence. Sequence parameters were as follows: repetition time/echo time (TR/TE) = 2500/30 ms, slice thickness = 3.5 mm, field of view (FoV) = 256 mm, flip angle (FA) = 90°, data matrix = 64 × 64, in-plane resolution = 3.5 × 3.5 mm, 46 slices, 10 min scan length. Four dummy scans were discarded to remove the impact of magnetization instability. A high-resolution (spatial resolution: 1 mm^3^) structural image was also acquired on the first day using a T1-weighted magnetization prepared rapid-acquisition gradient echo (MP-RAGE) pulse sequence.

### Data preprocessing

All preprocessing steps were performed using the Data Processing Assistant for Resting-State fMRI Advanced Edition (DPARSFA)^31^, which runs on Statistical Parametric Mapping 8 (SPM8) and the Resting-State fMRI Data Analysis Toolkit (REST)^32^. Data preprocessing included the following steps: realignment of all functional images using a six-parameter rigid body transformation (T_x_ = 0.04 ± 0.03 mm, T_y_ = 0.30 ± 0.14 mm, T_z_ = 0.21 ± 0.13 mm, R_x_ = 0.27 ± 0.13°, R_y_ = 0.06 ± 0.05°, R_z_ = 0.10 ± 0.05°); slice-timing correction to the middle slice of each volume; co-registration of the structural image (T1-weighted MPRAGE) to the mean functional image using a rigid-body transformation; segmentation of the transformed structural image into the gray matter, white matter, and cerebrospinal fluid (CSF); nuisance covariate regression of six head motion parameters, average white matter signals, CSF signals, and the global signal in native space; spatial normalization of the functional images to the Montreal Neurological Institute (MNI) stereotactic standard space; spatial smoothing of the functional images with a 6-mm full-width at half-maximum (FWHM) Gaussian kernel using the Diffeomorphic Anatomical Registration Through Exponentiated Lie Algebra (DARTEL) toolbox^33^; band-pass filtering (0.01–0.10 Hz) to reduce low-frequency drift and high-frequency physiological noise. The relationship between the framewise displacement^34^ and the following indices are summarized in the Supplementary material.

### Computation of functional connectivity

To calculate a functional connectivity matrix for each day, we defined the regions of interest (ROI) based on the Human Connectome Project (HCP)’s multi-modal parcellation version 1.0^35^ and the automated anatomical labeling (AAL) atlas^36^. Of the 360 cerebral cortical ROIs defined by the HCP, one ROI (rh.R_8BL) was excluded because the obtained mask contained only 2 voxels. In addition to the remaining 359 cerebral cortical ROIs, we included 40 limbic and cerebellar ROIs defined by the atlas, resulting in a total of 399 ROIs. For each ROI, we computed the average time course of the signal at voxels in the mask. For each day, we quantified functional connectivity between each pair of the 399 ROIs by the absolute value of the Pearson correlation coefficient between the two fMRI time-course signals^37,38^.

### Similarity of the functional connectivity across time

To calculate correlation of functional connectivity between days. Specifically, we first vectorized the functional connectivity between all pairs of 399 ROIs for each day into a 399 × 398/2 = 79,401 dimensional vector. Then, we computed the Pearson’s correlation coefficient between the two vectors for the corresponding days.

### Generalized Louvain method

The original Louvain method approximately maximizes the objective function (modularity) to partition the nodes in the given static network into communities. Communities are determined such that there are many edges or connections within each community and relatively few edges between communities^39^. The generalized Louvain method considers the edges across multiple inter-dependent days and optimizes the generalized modularity instead of separately optimizing the modularity for each day. In the present study, a day represents the static functional connectivity on one day. The strength of the connections between days and the spatial resolution parameter were set as default (i.e., $\omega$ = 1) and the resolution parameter (i.e., $\gamma$ = 1). We ran the algorithm 100 times and selected the community architecture yielding the largest generalized modularity value. As shown in the Results section, this procedure found four communities that are comparable with the communities in previous research^40–42^.

### Community labeling

First, we represented each community *i* (*i* = 1, 2, 3, 4) by its core members. The core members of the *i*th community were defined by the ROIs whose dominant community, that is the community to which the ROI belonged for the largest number of days under the given condition, was the *i*th community under both conditions. The relative overlap between the *i*th community and a community in the template communities defined by Yeo et al.^43^, *N*_*j*_ (*j* = 1, 2, …, 7), was defined as ${V}_{\left({C}_{i}\cap {N}_{j}\right)}/{\sum }_{l=1}^{7}{V}_{\left({C}_{i}\cap {N}_{j}\right)}$, where ${C}_{i}$ is the set of voxels belonging to a core member of the *i*th community, *N*_*j*_ is interpreted as the set of voxels belonging to mask ${N}_{j}$, and ${V}_{({C}_{i}\cap {N}_{j})}$ represents the number of voxels that belong to both ${C}_{i}$ and ${N}_{j}$. We then labeled each community *i* according to the community in the template that exhibited the largest overlap with the *i*th community.

### Calculation and statistical significance test of community size

We defined the community size for a day *t* as the number of regions included in the time-dependent community on day *t*, and tested the difference of average community size across the conditions. We also explored potential changes in the community size over time by including days as an additional independent variable. More specifically, we performed a general linear model in which practice condition (meditation condition vs. no-meditation condition; a categorical variable), community (visual network, SMN, FPN, and DMN; a categorical variable), and day (*t*: 1,2, …, 58; a continuous variable) were included as independent variables. We also included the interactions of these independent variables.

### Calculation and statistical significance test of community coherence

We defined the similarity of each community *i* (*i* = 1, 2, 3, 4) between day ${t}_{1}$ and day ${t}_{2}$ by the Jaccard index, i.e. ${J}_{(X, Y)}=\left|X\cap Y\right|/\left|X\cup Y\right|$, where *X* is the set of nodes in community *i* on day ${t}_{1}$ and *Y* is the set of nodes in community *i* on day ${t}_{2}$. Jaccard index ${J}_{(X,Y)}$ ranges between 0 and 1. One obtains = ${J}_{(X,Y)}$1 if and only if *X* and *Y* are exactly the same, and = ${J}_{(X,Y)}$0 if *X* and *Y* do not share any ROIs. For each of the four communities, we calculated the similarity between all pairs of 58 days, obtaining a 58 × 58 similarity matrix.

Given the similarity matrix for a community, we compared the coherence of the community within and across the practice conditions. Specifically, we used a permutation test, which is commonly used for testing the significance of single-subject research^44^. The permutation test consists of the following three steps. In the first step, we classified the pairs of days into two groups. The congruent group contained the pairs of days which both belonged to the meditation condition or the pair of days which both belonged to the no-meditation condition. In contrast, the incongruent group contained the pairs of days from the different conditions (i.e. one from the meditation condition and the other from the no-meditation condition). Because there were 18 meditation days and 40 no-meditation days, congruent and incongruent groups contained 933 and 720 pairs of days, respectively. In the second step, we computed the coherence of the community, which is a Welch’s *t*-value, by comparing the averaged similarity value between the congruent and incongruent groups. We then randomized the days by reassigning 18 uniformly randomly selected days to the fictive meditation condition and the remaining 40 days to the fictive no-meditation condition, and calculated the coherence for the randomized data. We repeated this procedure 10,000 times to obtain the null distribution of the coherence for the randomized labeling. In the third step, we assessed the probability of obtaining the coherence calculated on the basis of the true labeling of the days (i.e., meditation condition or no-meditation condition) or more extreme coherence under the null model in which the meditation condition was randomly assigned to individual days.

### Calculation and statistical significance test of flexibility

We defined the flexibility of a ROI under each condition using the inverse participation ratio (IPR)^45^. The IPR of a ROI under a condition is defined as

$$IPR = 1-\sum_{i=1}^{4}{\left(\frac{{I}_{i}}{I}\right)}^{2},$$

where *I* represents the number of days (no-meditation condition, 40; meditation condition, 18), and *I*_*i*_ represents the number of days in which the ROI belonged to community *i* (*i* = 1, 2, 3, 4). The IPR is equal to 0.75, which is the largest possible value, when a ROI belongs to all the communities with the same probability. In this case, the ROI is the most flexible in terms of the community membership. The IPR is equal to 0, which is the smallest possible value, when the ROI belongs to the same single community in all the days. In this case, the ROI is the least flexible. To investigate whether the meditation affects the community-wide flexibility of ROIs, for each community, we applied a paired sample *t*-test to test the mean difference in the flexibility between the two conditions. In this analysis, we defined each community *i* by its core members, i.e., the ROIs that belonged to community *i* as the dominant community under both conditions.

### Consent for publication

An earlier version of this article is present on bioRvix repository website and can be accessed on https://www.biorxiv.org/content/10.1101/664573v2. This article is not published nor is under publication elsewhere.

## Results

### Similarity of the functional connectivity across time

To examine variability of the functional connectivity over time, we calculated the correlation of functional connectivity between days (Fig. 1A). The correlation value ranged from 0.429 to 0.780, suggesting that the functional connectivity of a single person varies on a daily basis. This result is consistent with previous longitudinal scanning data from a single participant^29^.

### Finding community architecture

**Figure 2:**
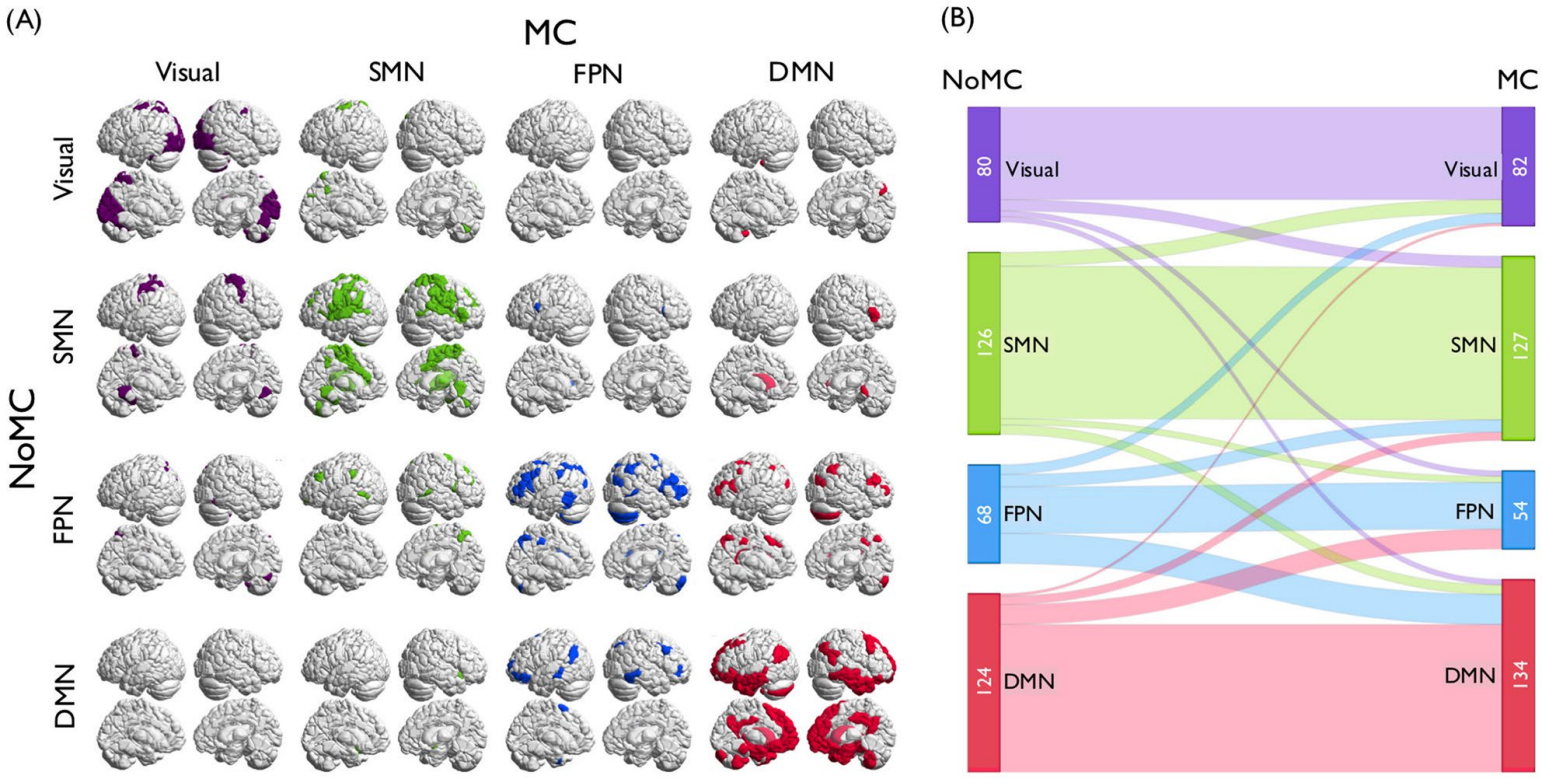
The change of dominant community affiliation between the no-meditation condition (NoMC) and the meditation condition (MC). (**A**) The shift of the dominant community allegiance of ROIs between the conditions. The schematic pictures of the brain on the diagonal show the ROIs that belonged to the same community with the highest probability across the two conditions. Those off the diagonal show the ROIs that belonged to different communities in the two conditions. (**B**) Quantitative description of dominant community shift across the conditions. While the ROIs were generally classified to the same community in both the MC and the NoMC, a large number of ROIs (i.e. 21 ROIs) that belonged to the FPN in the NoMC shifted to the DMN in the MC (row 3, column 4; other community pairs $\le 11$ ROIs).

To detect the community architecture in the time-varying functional connectivity across the 58 days, we applied a generalized Louvain method^46^ (Fig. 1B). To label the four communities detected in the current study, we assessed how these communities overlapped with the template communities defined by Yeo et al.^43^. As the result, we labeled the four communities as visual network (80.8% overlap), SMN (50.3% overlap), FPN (45.3% overlap), and DMN (58.4% overlap) (diagonal in Fig. 2A).

### Metrics that quantify the changes in community architecture

We examined the effect of meditation practice on intra-individual changes in the composition of the whole-brain networks with three metrics: *community size*, *community coherence*, and *flexibility*.

### Community size

The analysis showed a significant main effect of community (*F*_(3, 216)_ = 147.7, *p* < 0.001) while we observed no significant main effect for the condition (*F*_(1, 216)_ = 0.01, *p* = 0.907) nor for the day (*F*_(1, 216)_ = 0.00, *p* = 0.999). These main effects were qualified by a significant interaction between the community and the condition (*F*_(3, 216)_ = 3.03, *p* = 0.030), suggesting that meditation changed the community size differently across the communities. To explore which communities showed the differential effect, we took all the possible pairs of communities (e.g., FPN and SMN) and examined whether the effect of meditation on community size was different between the paired communities. The results showed that meditation condition only significantly interacted with the FPN and the DMN (*F*_(1, 112)_ = 4.54, *p* = 0.035; others, *F*_(1, 112)_ ≤ 2.64, *p* ≥ 0.107). The significant interaction indicates that meditation increased the community size of the DMN while it decreased the size of the FPN, although post-hoc analysis did not show statistically significant simple main effects of meditation condition either for the FPN (*F*_(1, 56)_ = 2.317, *p* = 0.134) or the DMN (*F*_(1, 56)_ = 2.515, *p* = 0.118). We also observed a significant interaction across the community, condition, and day (*F*_(3, 216)_ = 9.89, *p* < 0.001). To unpack the three-way interaction, we examined the interaction between the meditation condition and day separately for each community. The interaction between the meditation condition and the day was significant for the FPN (*F*_(1, 54)_ = 7.77, *p* = 0.007) and DMN (*F*_(1, 54)_ = 9.46, *p* = 0.003) but not for the visual network (*F*_(1, 54)_ = 3.52, *p* = 0.066) and SMN (*F*_(1, 54)_ = 0.40, *p* = 0.527). In the FPN, the day effect was significantly positive in the meditation condition (*F*_(1, 16)_ = 6.06, *p* = 0.026) and not in the no-meditation condition (*F*_(1, 38)_ = 0.25, *p* = 0.617) indicating that the community size of the FPN increased as the meditation practice progressed. In the DMN, on the other hand, the day effect was significantly negative in the meditation condition (*F*_(1, 16)_ = 6.98, *p* = 0.018) and not in the no-meditation condition (*F*_(1, 38)_ = 0.73, *p* = 0.400), indicating that the community size of the DMN decreased as the meditation practice progressed.

Figure 2A shows whether the ROIs stayed in the same community or changed to a different dominant community between the two conditions (i.e. represents the main community that a ROI belonged to under each condition). The on-diagonal brains show the ROIs that stayed in the same dominant community across the two conditions. The off-diagonal brains show the ROIs that belonged to different dominant communities between the two conditions. Consistent with the results, the figure shows that a large number of ROIs (i.e. 21 ROIs) that belonged to the FPN in the no-meditation condition shifted to the DMN in the meditation condition (row 3, column 4; other community pairs $\le 11$ ROIs). The shift in the dominant community affiliation between the conditions is quantitatively depicted in Fig. 2B.

### Coherence of community composition

**Figure 3:**
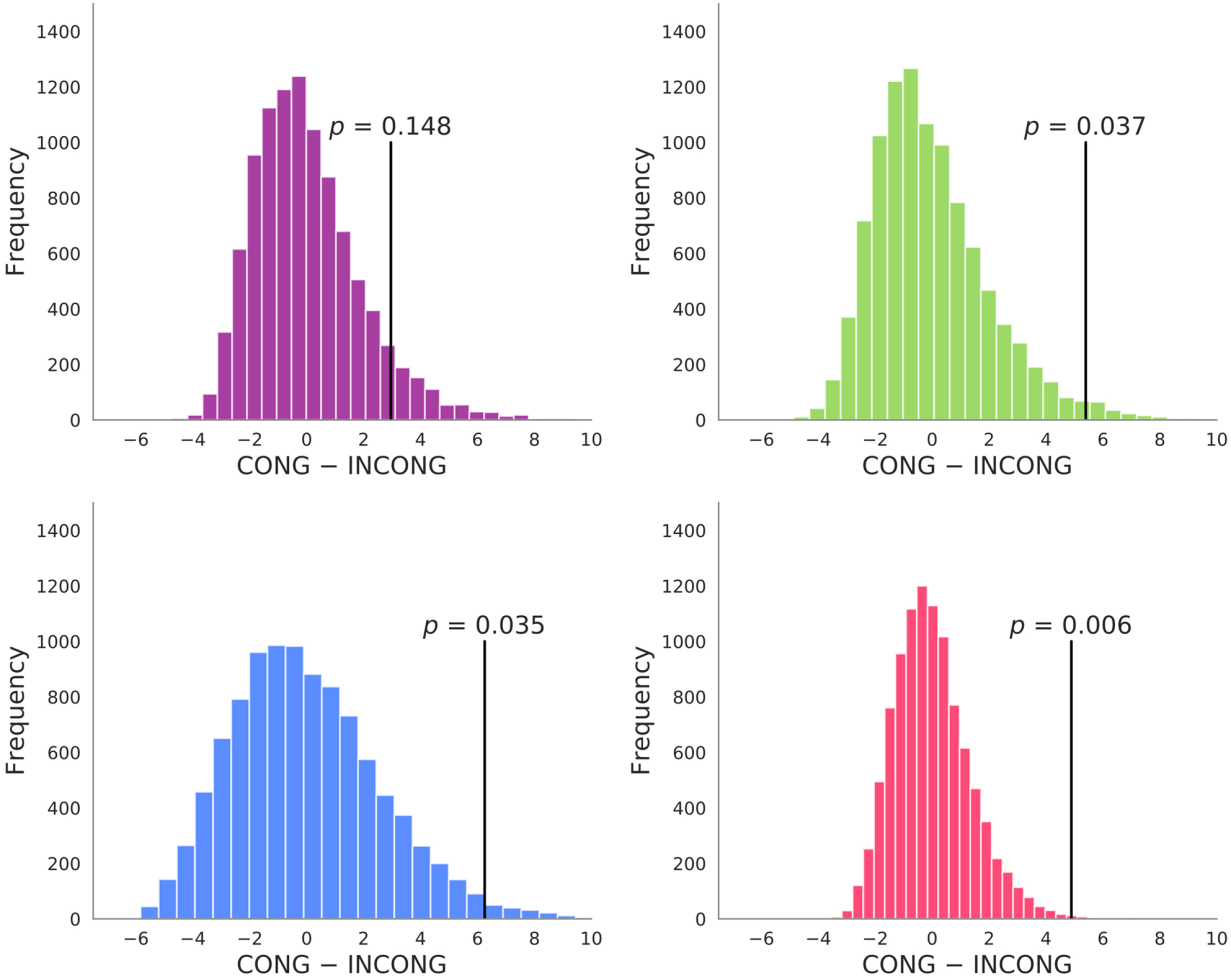
Coherence of the community within and across conditions. Coherence is a Welch’s t-value that represents the difference in averaged similarity value of a community architecture between two groups, i.e., the congruent and incongruent group. The congruent group contained pairs of days that both belonged to the meditation condition (MC) or the no-meditation condition (NoMC). The incongruent group contained the pairs of days from the different conditions (i.e. one from the MC and the other from the NoMC). The figure shows null distributions of coherence (distributions of coherence for permutated data); vertical lines represent the t-values observed with the true labeling and their corresponding *p*-values representing the probability of obtaining such observed t-values (or more extreme t-values) in the null distribution. Significant effect was found only in the DMN after FDR correction, which would be a direct consequence of our findings on the community size because a change in the community size implies a decrease in the coherence value.

The community size is one way of examining the change in the composition of the brain network across the two conditions. In fact, even if the relative community size is the same between the two conditions, the constituent ROIs of each community may be substantially different in the two conditions. Therefore, for each community, we examined the extent to which the community as identified by the set of ROIs comprising it is stable within each of the two conditions (community coherence). The results of the permutation test are shown in Fig. 3. For the DMN, the coherence of the community architecture within the same condition was larger than the coherence between the no-meditation condition and meditation condition (DMN, *p* = 0.006), which remained significant after correction of the false discovery rate (*FDR* = 0.05). This result indicates that the meditation practice has changed the community composition (i.e., the set of ROIs composing the community) in the DMN, which is a direct consequence of our findings on the community size because a change in the community size implies a decrease in the coherence value. For the other communities, there was no difference in the coherence (visual, *p* = 0.137; SMN, *p* = 0.030; FPN, *p* = 0.028), which implies that the sets of ROIs composing these network communities were not influenced by the meditation at a significant level.

### Flexibility of community allegiance

**Figure 4:**
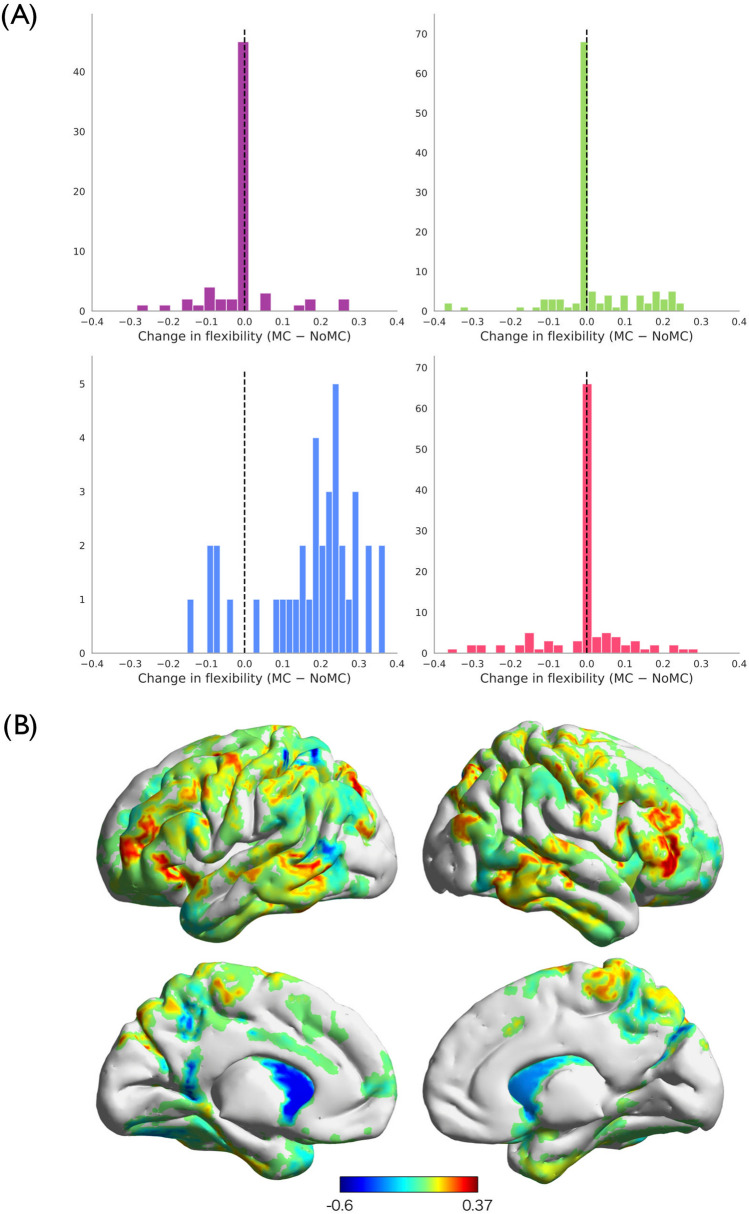
Changes in flexibility between two conditions. (**A**) Histograms for each community show the distribution of the values of ROIs that belonged to the corresponding community in both conditions. (**B**) Change in flexibility rendered on the cortical surface. Positive values mean the flexibility was higher in the meditation condition (MC) than the no meditation condition (NoMC). The ROIs that showed higher flexibility in the MC were in fronto-parietal network (FPN), whereas that showed higher flexibility in NoMC scattered in networks.

To assess experience-related changes in community allegiance of a ROI, we defined the flexibility of a ROI under each condition using the IPR (Derrida and Flyvbjerg^45^). The change in flexibility between meditation condition and no-meditation condition for individual ROIs belonging to each community (i.e. visual network, SMN, FPN, and DMN) is shown in Fig. 4A. Positive values mean that flexibility of the ROI increased as a consequence of the meditation. One-sample *t*-tests of the difference in flexibility between the two conditions for each community revealed that the meditation significantly enhanced flexibility of the ROIs in the FPN (Fig. 4B; mean = 0.17 ± 0.13, *t* = 7.334, *p* < 0.001). Other communities did not show a significant difference in flexibility of the ROIs (|mean| ≦ 0.02, SD ≧ 0.10, |*t*| ≦ 0.802). These results suggest that the meditation increases the flexibility of the FPN community, but not the visual network, SMN, or DMN. Table 1 summarizes the results.

**Table 1:** Summary of the meditation effect. *n.s.* indicates not significant.

Network	Size	Coherence	Flexibility
	The number of ROIs in the community	Change in the composition of ROIs in a community	Frequency with which ROIs change their community allegiance
Visual	*n.s*	*n.s*	*n.s*
SMN	*n.s*	*n.s*	*n.s*
FPN	NoMC > MC	*n.s*	NoMC < MC
DMN	NoMC < MC	NoMC $\ne$ MC	*n.s*

*MC* meditation condition, *NoMC* no-meditation condition.

## Discussion

Previous studies have provided evidence that focused attention meditation changes activation and connectivity patterns between specific brain regions^10,19–23^. Extending on this line of research, we employed a whole-brain graph theoretic analysis with a single-case experimental design using intensive longitudinal data to reveal that the meditation provokes the reconfiguration of the community architecture of the whole-brain functional network.

We found that the size of the FPN decreased and that of the DMN increased as a consequence of the meditation, although their size tended to return to the default size in the later period of experiment. The former result is consistent with the previous research in which experienced meditators showed increased functional connectivity of the PCC with the dorsal ACC and dorso-lateral PFC both during rest and meditation^19^ compared with novice meditators. The research proposed that the composition of these brain networks might have changed over time and become a new “default mode” that can be observed during meditation as well as during the resting state. The current observation that some ROIs shift from the FPN to the DMN after the meditation partially supports their hypothesis. Considering such consistency, the return of the community size in the later period might reflect insufficient effect of the practice because of habituation-derived lack of concentration.

The FPN also showed enhanced flexibility under the meditation condition. A previous study suggested that enhanced flexibility in the FPN may reflect an (initial) learning process of a task^47^. Accordingly, although the previous study used a different task with a different time scale, i.e., Bassett et al.^48^ observed the change in the flexibility within a few hours of motor-task training, the increased flexibility in the FPN in the current study may indicate that some form of learning process is operative in the meditation (e.g., how to control breathing, attention, bodily sensations etc.). Also, previous research proposed that the ROIs in the FPN integrate and modulate other networks in response to varying task demands^48–50^. These findings are consistent with the observations from the current study; the FPN’s enhanced flexibility as well as its integration into the DMN under the meditation condition (Figs. 2B and 4) may indicate that meditation practice would increase the FPN’s efficacy to inhibit the DMN during a situation on which individuals have to focus. Future research should directly examine the relationship between the reorganization and reconnection of the FPN and the DMN, and the positive effects of meditation such as improved concentration or emotion regulation.

The present study demonstrated the value of a single-case experimental design with intensive longitudinal data. It allows us to detect intra-individual changes in the whole-brain network composition without being influenced by the large heterogeneity of individuals’ brain functional networks^25^. Previous studies on meditation heavily relied on the expert-beginner and/or pre-post comparison design^1^. Future research should be encouraged to adopt the single-case research design more frequently to seek further insights into intra-individual changes in patterns of brain networks as a consequence of meditation. One obvious limitation of the current research design is that the data were collected from a single participant, which makes it impossible to examine potential individual differences in our findings. However, although research in cognitive neuroscience typically collects data from multiple participants, for the majority of studies, their main focus is on the aggregated pattern of the brain activation/connectivity (but see person-centered research^24^), and individual differences have been typically treated as random noise (sampling error). Therefore, in our view, this limitation is superseded by the strength of the current design: sensitivity to the nuanced intra-individual changes in brain signals and functional connectivity. Nevertheless, the potential of the current intensive longitudinal design would be considerably improved by data obtained from multiple participants in future studies. Another limitation of the present study is that the participant performed meditation practice only for three months, which is considerably shorter than the previous studies with experts (e.g., more than one year of regular practice)^10^. In addition, due to the fact that the intervention was planned after the scanning session had started, meditation practice was embedded in the latter half of the period. This design issue makes it difficult to completely distinguish the exact effect of mindfulness from the general time course effect, although there is little evidence that repeated scans would change the nature of functional connectivity networks^29,51^. Future research should collect data for a more prolonged period of time to examine how the progress of practice induces long-term changes in the community architecture.

## Supplementary information

Supplementary Information.

## Data Availability

The data that support the findings of this study are available from the corresponding author upon reasonable request.
