# Supplementary material for: Focused attention meditation changes the boundary and configuration of functional networks in the brain
Source: Sci Rep. 2020 Oct 28;10:18426. doi: 10.1038/s41598-020-75396-9 (PMC7595086; doi:10.1038/s41598-020-75396-9)
Supplement: Supplementary file 1 — Supplementary Information. [file 41598_2020_75396_MOESM1_ESM.docx]

**Title**: Focused attention meditation changes the boundary and configuration of functional networks in the brain.

**Authors**: Shogo Kajimura^1,*^, Naoki Masuda^2^, Johnny King L. Lau^3^, Kou Murayama^3^

^1^Faculty of Information and Human Science, Kyoto Institute of Technology, Kyoto, Japan; ^2^Department of Mathematics, University at Buffalo, State University of New York, USA; ^3^Department of Psychology, University of Reading, UK

**Supplementary results**

We calculated the framewise displacement (FD) based on the Power et al. (2012) and found that it was small throughout the study (mean = 0.103, SD = 0.015). We checked the relationships between the FD and main findings regarding flexibility, community size, and community coherence like below.

Flexibility

We could not calculate correlation between the FD and flexibility because they have different dimensions; the FD is date-level (58 values), whereas the flexibility is ROI-level (399 values). So, we indirectly assessed the influence of the FD to the current result of flexibility (and the others) by testing whether the FD was significantly different between conditions (MC vs. NoMC). The difference was not significant (*t* = -0.373, *p* = 0.711), indicating that the significant results we found were not due to the influence of FD.

Community size

For each community, we calculated Pearson’s correlation of the FD and community size. The significance was estimated by the permutation test that is described in the manuscript (p13, line2-). In brief, we obtained a null distribution of the correlation by shuffling the FD and calculated the correlation for 1,000 times. The correlation of the true FD and community size would be significant if the probability of obtaining the correlation is considerably small (i.e., *p* < 0.05). The correlation of the FD and community size was not significant (|*rs*| $\leq$ 0.220, *ps* $\geq$ 0.108), indicating that the significant result we found in the community size was not due to the effect of FD.

Coherence

The coherence was similarity-based index: similarity of community composition between condition-congruent days (congruent group) vs. similarity of community composition between condition-incongruent days (incongruent group). So, we first averaged the similarity of congruent and incongruent groups for each community and day, and then calculated correlation with the FD. The significance was estimated by the permutation test described above. The correlation of the FD and the similarity was not significant in congruent groups (|*rs*| $\leq$ 0.200, *ps* $\geq$ 0.136) or (|*rs*| $\leq$ 0.194, *ps* $\geq$ 0.128), indicating that the significant result we found in the community coherence was not due to the effect of FD.
